# A Curious Case of Acute Acalculous Cholecystitis

**DOI:** 10.7759/cureus.14948

**Published:** 2021-05-10

**Authors:** Ruqaiyyah Hamid, Rasiq Zackria, Jyotsna S Sharma

**Affiliations:** 1 Internal Medicine, Dow International Medical College, Karachi, PAK; 2 Internal Medicine, University of California Riverside, Riverside, USA; 3 Internal Medicine, University of California Los Angeles, Los Angeles, USA

**Keywords:** acute acalculous cholecystitis, cholecystitis, viral hepatitis, gallbladder, inflammation of gallbladder

## Abstract

Acute acalculous cholecystitis is inflammation of the gallbladder without any evidence of gallstones. Although acalculous cholecystitis is less common than its calculous counterpart, it can be fatal if not treated. It is essential to rule out the cause of acalculous cholecystitis to aid in the treatment and management of the patient. We present a case of acalculous cholecystitis wherein a comprehensive workup found the etiology to be viral. Albeit rare, hepatitis A and cytomegalovirus can be causes of acute cholecystitis. Both viruses were observed simultaneously in this patient, proving it to be a unique case. This early diagnosis allowed conservative management of the patient, sparing him from unnecessary surgical intervention.

## Introduction

Inflammation of the gallbladder without any evidence of gallstones is known as acute acalculous cholecystitis. While it is relatively less common to its calculous counterpart, the associated mortality is significantly higher. Acute acalculous cholecystitis has many etiologies, including viruses, and determining the etiology of acute acalculous cholecystitis can aid in the management of the patient. Hepatitis A virus (HAV) generally causes a self-limited infection of the liver, and most cases of hepatitis A resolve spontaneously. While hepatic and extrahepatic complications have been recognized for HAV, acute acalculous cholecystitis is a rare complication of acute viral hepatitis [1]. Also rare is acute cytomegalovirus (CMV) infection as an etiology for acute acalculous cholecystitis [2]. Herein, we report a case of acute acalculous cholecystitis due to acute HAV infection with concurrent acute CMV infection.

## Case presentation

**Figure 1:**
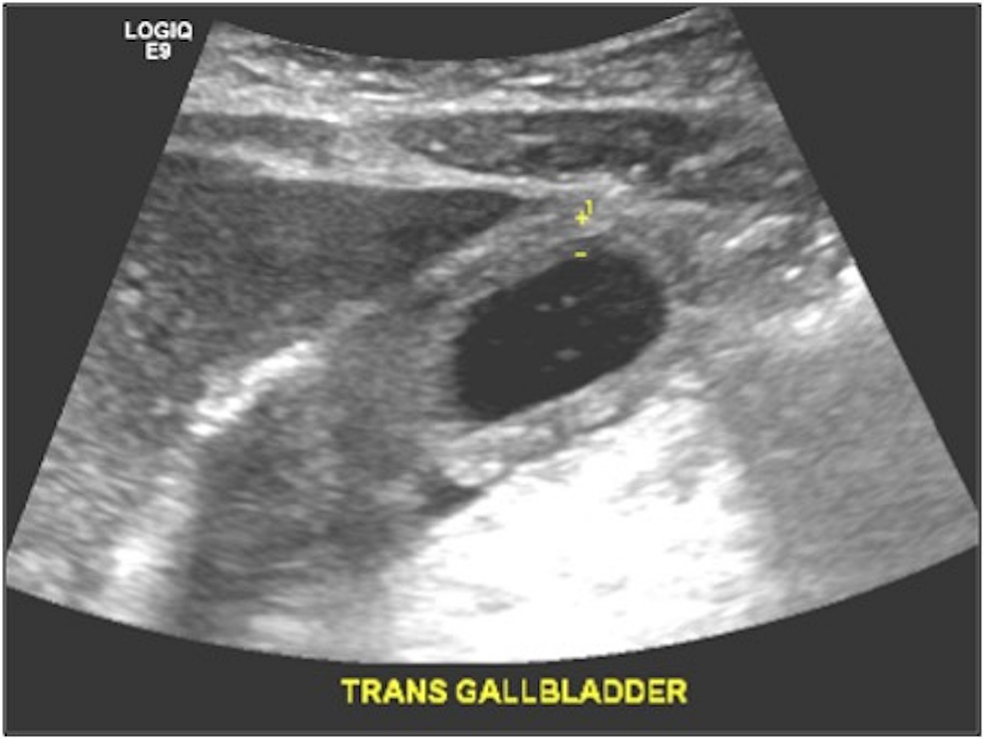
Gallbladder ultrasound showing no evidence of gallstones, but demonstrating gallbladder wall thickening, and pericholecystic fluid.

A 37-year-old man with a five-year history of IV heroin abuse (sober for 10-years) presented with worsening epigastric pain, loss of appetite, nausea, and dark urine of one week duration. He did not have a primary care physician, and no other medical history was known. He denied any other symptoms and alcohol abuse. Vital signs were within normal limits. Physical exam was pertinent for exquisite tenderness to light palpation in the epigastric and right upper quadrant of the abdomen; there was no evidence of jaundice. Laboratory data revealed markedly elevated liver enzymes with alanine aminotransferase (ALT) of 3,153 U/L, aspartate aminotransferase (AST) of 2,489 U/L, and total bilirubin of 3.3 mg/dL, that was predominantly conjugated. Other laboratory studies, including complete blood count, serum lipase, and blood alcohol level, were within normal limits. Abdominal ultrasonography (Figure 1) was remarkable for a positive sonographic Murphy’s sign and showed marked gallbladder wall thickness of 6 mm (normal ≤ 3 mm) with pericholecystic fluid and no biliary calculi or sludge.

Although the predisposing cause was not clear, a working diagnosis of acalculous cholecystitis was formulated. Initial management with intravenous hydration, analgesics, and antibiotics was associated with a mild improvement in symptoms. However, the significantly elevated transaminase levels raised suspicion for a primary hepatic etiology. Viral serologies were obtained, which revealed positive IgM for HAV and CMV, consistent with an acute infection. The patient was treated with supportive therapy, antibiotics were discontinued, and the tentative plan of laparoscopic cholecystectomy was shelved in favor of conservative management of his coinciding acute viral syndromes. By day 4 of the hospitalization, the patient had shown remarkable improvement, with down-trending liver enzymes. He was subsequently discharged to the rehabilitation program on hospital day 5 in stable clinical condition.

## Discussion

Acute acalculous cholecystitis is described as gallbladder inflammation without evidence of a stone and is usually encountered in critically ill, hospitalized patients. It makes up for 5%-10% of cases of all cholecystitis [3]. It is frequently associated with gangrene, perforation, and empyema, with a 10%-50% mortality rate in affected patients [2]. The risk factors and infections predisposing to acute acalculous cholecystitis are extensive but include CMV, EBV, HAV, and HBV [2]. Being immunocompromised, especially due to HIV infection, can increase the risk of contracting acalculous cholecystitis [4].

Clinical symptoms include fever, nausea, jaundice, right upper quadrant pain, and a positive Murphy’s sign. The diagnosis is suspected on clinical presentation, and an ultrasound can be used for confirmation due to its high sensitivity, specificity, and accuracy. Laboratory data may show increased liver transaminases, total bilirubin, and alkaline phosphatase; however, normal levels do not rule out the disease. Ultrasonographic criteria for diagnosis include gallbladder distention, gallbladder wall thickening greater than 3.5 mm, no evidence of biliary sludge, no dilation of the intra- or extrahepatic bile ducts, and presence of pericholecystic fluid [5].

HAV-induced acute acalculous cholecystitis has been rarely reported in the adult population. While the occurrence of acalculous cholecystitis during an acute HAV infection is uncommon, it has been well documented in the pediatric population, especially in developing countries with a higher incidence of HAV infection [6]. The pathophysiology of acalculous cholecystitis during acute viral hepatitis is unclear, although theories suggest hypoalbuminemia, an extension of the hepatic inflammation, and elevated portal pressure as a potential etiology for the gallbladder wall edema [7]. Acute acalculous cholecystitis as a result of HAV infection is transient and gradually improves when viremia becomes low; gallbladder wall thickness returns to usual size during this time as well, and hence, surgical intervention is generally not required [8].

The case presented here is of an adult patient who presented with clinical symptoms consistent with acute cholecystitis, which was confirmed to be acute acalculous cholecystitis with the ultrasonographic examination. HAV infection was suspected and confirmed serologically due to the elevated liver enzymes. Incidentally, we found an acute CMV infection. Few cases have reported CMV-associated acalculous cholecystitis; however, these have been primarily in patients with acquired immunodeficiency syndrome or an immunocompromised state due to transplant [9]. While acute acalculous cholecystitis due to CMV is generally encountered in immunocompromised individuals, it is rare to encounter an immunocompetent patient with CMV-induced cholecystitis. Furthermore, concomitant infection with acute HAV and CMV as the etiology is rare. Since our patient did not have an immunocompromised state and demonstrated a quick recovery with conservative treatment, we focused on his HAV as the primary culprit. As HAV infection generally symbolizes a benign course, neither antibiotic treatment nor surgical intervention was needed to manage this patient.

## Conclusions

Acute acalculous cholecystitis is a rare complication of acute viral hepatitis. Also rare is an acute CMV infection as an etiology for acute acalculous cholecystitis. While CMV should be considered in patients as the primary etiology in immunocompromised patients, HAV infection should be widely considered a cause of acute acalculous cholecystitis in otherwise healthy adult patients. Due to the benign nature of the virus, these patients could be treated conservatively, avoiding unnecessary invasive procedures.
